# A Conversation with Fay Probert

**DOI:** 10.1021/acscentsci.3c00514

**Published:** 2023-05-01

**Authors:** Rachel Brazil

In 2015, after earning a bachelor’s degree in mathematics and a PhD in analytical chemical biology, Fay Probert started looking for ways to unite the two. Now a Dorothy Hodgkin Career Development Fellow at the University of Oxford—specifically, at Somerville College—she has combined analytical chemistry with machine learning to create diagnostic tools based on nuclear magnetic resonance (NMR) spectroscopy. Probert simply pops a sample of blood plasma or urine into an NMR machine, and her algorithms isolate the signals from an array of small-molecule metabolites to produce a metabolic fingerprint that can be used to diagnose disease.

**Figure:**
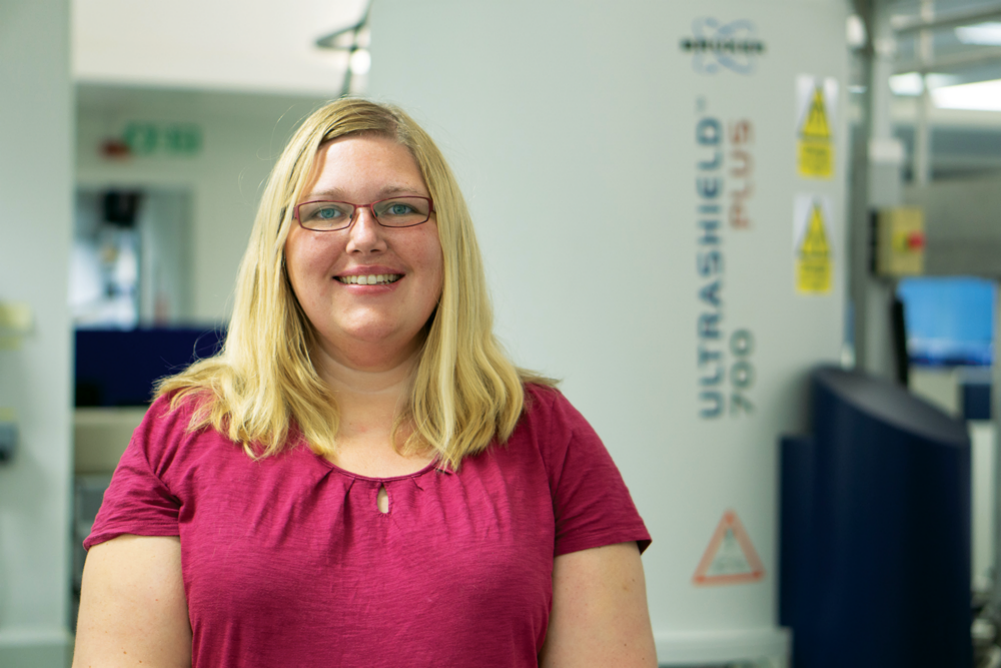
Credit: Jack Evans

Probert is working with colleagues, including Oxford oncologist James Larkin, on a metabolic fingerprint-based assay for diagnosing the type and aggressiveness of cancer in people presenting nonspecific symptoms. Similarly, in collaboration with Oxford’s Daniel Anthony, Probert has developed a diagnostic test for multiple sclerosis that can detect a person’s stage of disease. The cancer test is the focus of Oxomics, Probert and Larkin’s recently formed spin-off company; the multiple sclerosis test was licensed to the diagnostic company Numares Health in 2021 for further development and commercialization.

Probert is also using NMR metabolomics to better understand the chemistry of small-molecule pathways associated with disease and particularly the chemical processes associated with inflammation in the brain. She hopes the work will ultimately lead to improved treatments.

Rachel Brazil spoke to Probert about the disciplines that coalesce in her research and the role that machine learning could play in medicine. This interview was edited for length and clarity.

## What prompted your move from mathematics to analytical chemistry?

I picked maths as an undergraduate because I just thought it was beautiful. I wanted to do the most difficult thing I could think of. I did my undergraduate dissertation on modeling the hepatitis C virus, which I really enjoyed, and then, when I was finishing my undergrad degree, I wanted to do something that would hopefully help someone in a clinical setting.

I started to look around at courses that combined maths and chemistry and biology, and that’s how I ended up doing this multidisciplinary MSc in mathematical biology and analytical chemistry. And it’s where I first learned about NMR spectroscopy and fell in love with it, which led me to choose a PhD in NMR.

## What problems are you trying to solve?

All the work we do is informed by real clinical questions. So we’re not really interested in doing things like identifying a healthy person versus somebody with a late-stage cancer. That isn’t the real challenge, right?

It’s the patients that turn up to a GP clinic and have some symptoms that the GP thinks might be cancer, but they can’t say which type of cancer from any of the symptoms. These are the people who usually turn up to clinic—unfortunately as an emergency presentation, when of course the chances of survival are much lower.

Currently, with multiple sclerosis, the way to diagnose disease progression is from an expert neurologist, armed with a whole range of information—MRI scans and clinical information—and it can take some time to finalize that diagnosis. For cancer, a full-body CT scan is expensive, and we don’t have enough scanners and people across the country to interpret those scans. So our metabolomics test is sort of a way to triage those patients.

## Why use NMR spectra to provide this diagnostic information?

The first advantage of NMR is that the sample preparation is quite simple. We don’t have to filter the samples or isolate the specific molecules we want to measure. We can suppress any signals we are not interested in using a particular pulse sequence in the NMR. We measure just the small molecules and the lipoproteins—all in a single experiment that just takes a few minutes. The lipoproteins—the particles that carry cholesterol through the bloodstream—are particularly involved in inflammation, so they seem to be very useful biomarkers for a lot of the autoimmune inflammatory diseases we are interested in.

NMR is extremely information rich, so it gives you a biological or metabolic fingerprint of a person at a given point in time, and then the challenge is, how do you extract the important information from that fingerprint? For that, we use pattern recognition and machine learning methods to build equations that allow us to diagnose patients based on their metabolic fingerprint.

## What do you learn from these metabolomic fingerprints?

For blood samples, we’re measuring things like sugars, amino acids, and ketones, but also lipoproteins and fatty acids. [A metabolic fingerprint] from a person with a disease can give us a novel biomarker, and that’s when the chemical and biological knowledge comes in.

We really want to understand the biology behind this: What metabolite pathways are being perturbed here? How does that affect the whole chemistry within the cell? From that we develop hypotheses and develop new experiments to probe those pathways in more and more detail—and potentially find new drug targets in the future.

## What can you currently diagnose using your tests?

At this stage, the cancer test is just focusing on the nonspecific signs of any cancer and on whether the cancer has metastasized, but our hope with the spinout is that we will expand into specific cancers. The goal is for the first machine learning algorithm to tell you “cancer” or “no cancer.” If it’s cancer, does it look like a lung cancer? Does it have the same profile as a colon cancer?

And for our multiple sclerosis test, we can tell with an accuracy of 91% whether someone has transitioned to the later, secondary progressive stage of disease. There is no other blood test that is able to do that. We’re developing that for clinical use with a company called Numares, in Regensburg, Germany.

It’s been a really good experience for me to learn about developing my research into an approved diagnostic test, because that’s the dream of anyone who does any kind of work in science as a whole. Everyone wants their research to, at some point, help someone.

*Rachel Brazil is a freelance contributor to*Chemical & Engineering News*, the independent news outlet of the American Chemical Society.*

